# PCR primer design for mitochondrial cox-1 gene from Clinostomum complanatum towards diagnosis

**DOI:** 10.6026/97320630018831

**Published:** 2022-09-30

**Authors:** Monica Misra, Bhumika Chauhan, Km Deepika, A Madhuri, Bindu Sharma

**Affiliations:** 1 Department of Zoology, Acharya Narendra Dev College, University of Delhi, India; 2 Laboratory of Molecular Parasitology, Department of Zoology, Chaudhary Charan Singh University, Meerut, India; 3 Government Degree College for Women, Begumpet, Hyderabad, India; 4 Laboratory of Molecular Parasitology, Department of Zoology, Chaudhary Charan Singh University, Meerut, India

**Keywords:** Clinostomum complanatum, zoonotic, primer, design, cox-1, gene, DNA

## Abstract

Metacercariae of Clinostomum Leidy, 1856 are frequently encountered in freshwater fish. Clinostomum complanatum is a digenetic zoonotic parasite harbouring the intestine and body cavity of the fishes. 19 human incidences of Clinostomum complanatum infection have been reported to cause pharyngitis and lacramalitis from Japan, Thailand and Korea. Hence, adequate yet effective diagnosis is an issue. Designing primers used in the amplification of genes with adequate specificity and efficiency is of help in diagnosis. Hence, we describe primer design for cox-1 gene from the helminth parasite, Clinostomum complanatum parasitizing the intestine of fish Channastriata (Snakehead murrel). Thus, these designed primers set will be of further use in the wet lab for amplification of concerned gene or DNA fragment.

## Background:

Aquaculture has functioned as a pre-eminent key element in the economy of India. The increased incidences of parasitism and disease outbreaks have negative impacts not only on the fish production, but it also leads to economic losses [1, 3]. Clinostomum complanatum, is a digenetic trematode parasite of zoonotic importance that has been reported in various regions infecting the human population thus causing severe infections. Exploring and understanding the genome is the most prominent methodology for disease control. Till today many drugs and vaccines have been developed to minimize the parasitic infections caused by parasites thus preventing deaths caused by them. With the advent of high output technologies computer aided biology has played a significant role in understanding the genome, gene expression studies, physiochemical aspects of proteins, designing potent target drug molecules via docking and revealing the host–parasite interactions [4]. PCR based primers is helpful in this context. Hence, we describe primer design for cox-1 gene from the helminth parasite, Clinostomum complanatum parasitizing the intestine of fish Channastriata (Snakehead murrel).

## Methodology:

Retrieval of the sequence: We used the mt gene (cox-1) of C. complanatum genome. The sequence was retrieved from NCBI (National Centre of Biotechnology Information) database (http://www.ncbi.nlm.nih.gov) under accession number ON796499.

### Primer designing:

Primer designing was performed using the computational tools at NCBI.

## Results and Discussion:

Ten pair of primer sets was designed using computational tools (Figure 1 and 2 - see PDF). The average length of primer varies between 20-22 nucleotides. Usually, primer less than size 18 nucleotides is not considered an ideal primer as it cannot anneal with the genome of the target organism thus, unsuitable for use in wet labs. The GC content ranged from 47-55%. A high GC content requires a higher temperature to dissociate thus, the chances of amplification of gene is high [2]. The average temperature (Tm) was ranged between 57-60 degrees which is usually considered as ideal temperature. Thus, the designed primer sets fulfills the criteria of ideal primers for amplification of genes.

## Conclusion:

We illustrate the designing of 10 sets of primer pairs using the NCBI primer design tool. Thus, these designed primers set will be of use in the wet lab for amplification of concerned gene or DNA fragment.
